# Mechanical properties and acoustic emission characteristics of mixed granite after different numbers of freeze‒thaw cycles

**DOI:** 10.1038/s41598-024-65008-1

**Published:** 2024-06-18

**Authors:** Jinpeng Cao, Jun Hu, Xinrong Wang, Bin Yang, Zhiguo Xia, Hukun Wang, Linbin Zhang

**Affiliations:** 1 School of Civil Engineering, University of Science and Technology Liaoning, Anshan, 114051 China; 2 School of Mining Engineering, University of Science and Technology Liaoning, Anshan, 114051 China; 3 Ansteel Mining Engineering Corporation, Anshan, 114051 China

**Keywords:** Engineering, Civil engineering

## Abstract

The mechanical properties of rocks in cold regions undergo significant changes as a result of decades of freeze‒thaw cycles with seasonal variations, which can lead to a series of geological disasters, such as collapse. This study investigates the evolution of the mechanical characteristics and internal progressive damage characteristics of mixed granite under freeze‒thaw cycling and axial loading. By measuring the mass, wave velocity, and uniaxial compressive strength of rock samples and combining these metrics with acoustic emission (AE) characteristics, the physical and mechanical properties and microfracture development of mixed granite after different numbers of freeze‒thaw cycles were investigated. The results indicate that as the number of freeze‒thaw cycles increases, the longitudinal wave velocity, uniaxial compressive strength, and elastic modulus of the mixed granite decrease nonlinearly, while the peak strain gradually increases. Combined with the stress‒strain curve, the AE characteristics can be divided into four stages. As the number of freeze‒thaw cycles increases, the AE cumulative count decreases, and the AE counts of the four stages are different. The low-frequency-high-amplitude signals first increases and then tends to stabilize, and they only appeared in the third and fourth stages. At the same time, the proportion of the low-frequency ratio gradually increases, and the proportion of the high-frequency ratio decreases. In addition, based on the rise time/amplitude (RA) and average frequency (AF) characteristics and failure modes, it was found that the internal crack types of mixed granite transition from shear cracks to tensile cracks, among which tensile cracks play a crucial role in rock failure.

## Introduction

In recent years, the development of mineral resources and infrastructure construction have gradually expanded to high-altitude and cold regions^1^. As the seasons change, the shallow rock masses on the Earth's surface undergo irreversible cumulative damage under freeze‒thaw cycles, which may lead to ice wedging debris flows^2,3^, and even result in subgrade deformation^4^, instability and landslides of rock slopes^5,6^, and collapse disasters^7,8^ among other engineering disasters. When the temperature is lower than zero, the water in the pores and fractures of rock undergoes water–ice phase transition. The expansion force further expands the rock fractures, increasing the pore volume by approximately 9%^9,10^. As the temperatures rise, the ice melts into water, which further seeps into the expanding cracks. As a result, the next freeze‒thaw cycle progressively exacerbates the degradation of rock integrity and increases the risk of geological disasters. Therefore, it is of great theoretical significance to study the evolution and deterioration mechanism of rock mechanical characteristics under freeze‒thaw cycles for the stability of geological engineering in cold regions.

Research on the quasistatic physical and mechanical properties of rocks under freeze‒thaw cycles has achieved fruitful results^11,12^. These studies mainly focus on the effects of rock type^13^, water content^14^, loading^15^, and the number of freeze–thaw cycles^16^ on rock properties. The compressive strength, elastic modulus, and Poisson's ratio of sandstone with the same water content gradually decrease with the increase of the number of freeze‒thaw cycles, and the degree of decrease follows the exponential decay^17–19^. The freeze–thaw failure of rocks depends on the distribution of natural fractures, and almost all mechanical parameters decrease with increasing freeze–thaw cycles^20,21^. Representative studies are as follows: Wang et al.^22^ tested the static and dynamic mechanical properties of freeze-thawed rocks, and found that the freeze–thaw cycle had a detrimental effect on the mechanical properties of most of the sandstones, with the development of fissures, volumetric expansion, and weakening of cementation occurring. Shi et al.^23^ obtained the stress‒strain curves of red sandstone after different numbers of freeze‒thaw cycles by uniaxial and triaxial compression tests. The deterioration degree of the rock samples was quantified in terms of different mechanical parameters, such as the peak stress, elastic modulus and Poisson’s ratio. From the test results, each mechanical parameter was reduced, and the plastic damage after the peak stress was more obvious.

AE monitoring technology, as an effective method to capture rock microfracture signals in real time^24–26^, has been widely used in the field of rock mechanics. Zhang et al.^27^ studied the compression-induced damage mechanism of intact marble by AE monitoring. The AE monitoring technique has also been used in many studies of damage after freeze‒thaw cycles. Zhang et al.^28^ conducted shear tests on fractured rocks subjected to different numbers of freeze‒thaw cycles and found that the strain area of the samples increased with the increase in the number of freeze‒thaw cycles and that the samples were more prone to failure along the shear plane. Wang et al.^29^ also conducted a multifaceted AE study on defective granite after different numbers of freeze‒thaw cycles. They found that the AE counts and energy curves jumped during uniaxial compression by analyzing the AE signals and that these jumps were in line with the stress drops on the macroscopic stress‒strain curves. Furthermore, they also analyzed the AE spectra and concluded that the high amplitude-low frequency signals were best for predicting brittle fracture. Liang et al.^30^ conducted AE tests on saturated sandstone after freeze‒thaw cycles and found that the higher the number of freeze‒thaw cycles, the shorter the time it takes for the rock to reach the accumulated damage threshold. Wang et al.^31^ studied the deterioration properties of rocks with the help of AE technology and found that the AE event rate of sandstone gradually changed from the "V" type to the "U" type. Zhou et al.^32^ used real-time AE technology to reveal the damage characteristics of rocks under the action of 0 and 25 freeze‒thaw cycles, applied the critical slowing theory to calculate the AE autocorrelation coefficients and variances, and explored the characteristics of the damage precursors of rocks under the action of freeze‒thaw cycles.

The above research is of great significance for utilizing AE technology to analyze the microscopic characterization and damage patterns of rock samples during freeze–thaw processes. However, existing research on the macroscopic mechanical characteristics and AE characteristics of freeze-thawed granite is relatively dispersed. Therefore, this paper focuses on the mixed granite of an open-pit mine in Anshan, Liaoning Province as the research object. It conducts freeze–thaw cycle tests and uniaxial compression tests, monitors the AE characteristics during the compression deformation process of the rock samples, analyzes the degradation laws of physical and mechanical parameters, explores the relationship between AE parameters and changes in the internal mesoscopic structure of the rock, and comprehensively analyzes the damage evolution laws of mixed granite after the freeze–thaw cycle from multiple perspectives. It provides a scientific basis for the safety and stability of cold region mine engineering.

## Materials and methods

### Rock sample preparation and test equipment

**Figure 1:**
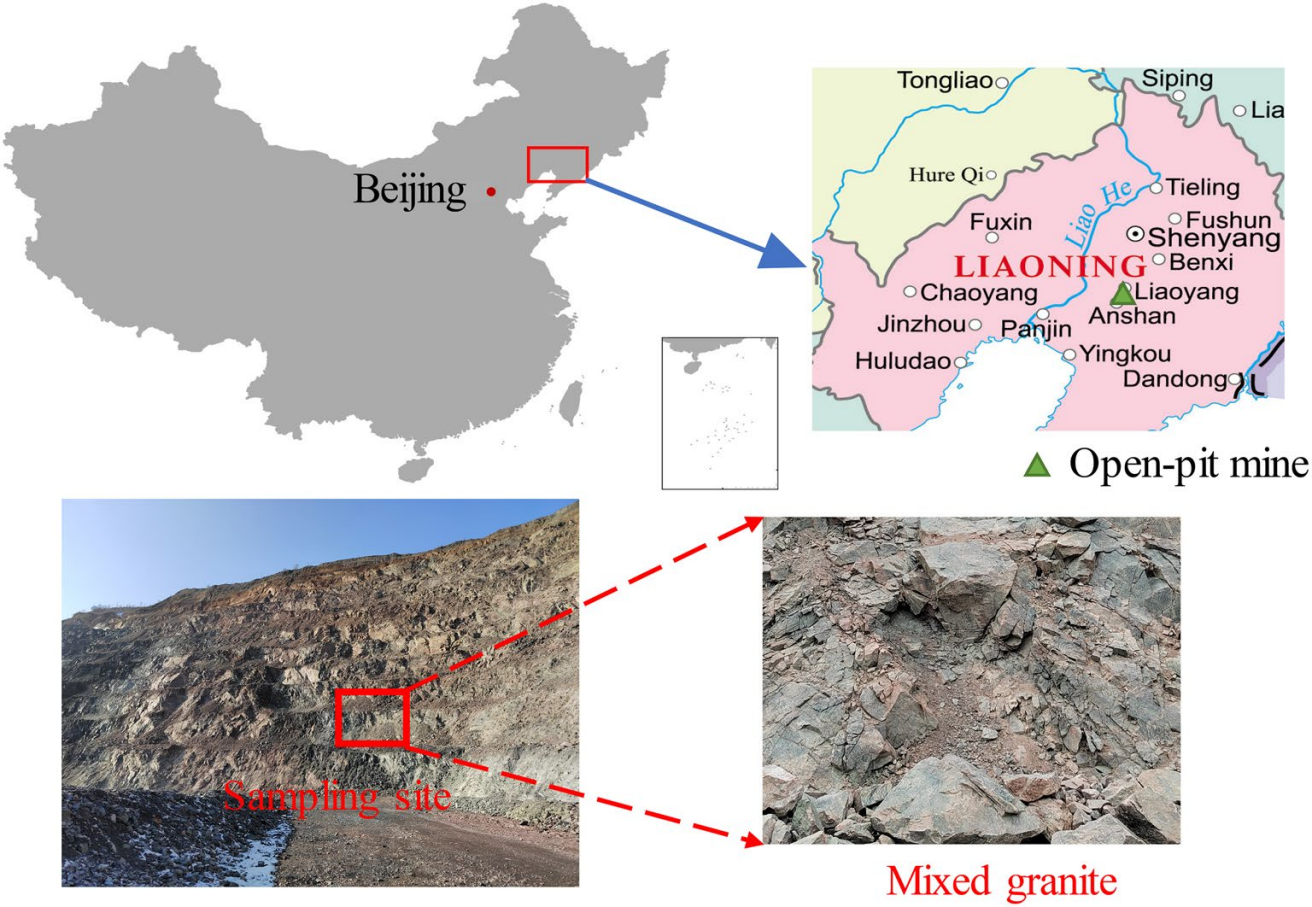
An open-pit mine in Liaoning Province.

The rock blocks were extracted from the slope of an open-pit mine in Anshan city, Liaoning province (Fig. 1). This area belongs to the seasonally frozen soil zone, characterized by an annual minimum temperature of − 21 °C and a maximum temperature of 36 °C. Mixed granite rock samples were taken from unblemished rock mass with flesh-red and pink surfaces, hard texture, and a more complex composition than ordinary granite, with amounts of marble, magnetite, quartzite, hornblende, and other dark rock residues. The average inherent natural density is 2660 kg/m^2^. Subsequent to core drilling, a standard rectangular sample measuring 50 mm × 50 mm × 100 mm was processed. After eliminating rock samples with obvious appearance defects, we selected rock samples with similar wave speeds using sonic velocimetry and finally identified 15 rock samples for freeze–thaw testing, and its physico-mechanical parameters are shown in Table 1.

**Table 1:** Physical and mechanical parameters of rock specimens.

Rock type	LWH/mm	*m*_*d*_*/*g	*m*_*s*_*/*g	*ρ*_*d*_*/*g cm^−3^	*e*_*0*_	*σ*_*uc*_*/*MPa	*E*_*0*_*/*GPa
Mixed granite	50 × 50 × 100	604.85	606.16	2.42	0.00524	130.67	28.79

*LWH*‒specimen size; *m*_*d*_*, m*_*s*_‒the dry and saturated mass; *ρ*_*d*_‒dry density; *e*_*0*_‒initial void ratio; *σ*_*uc*_‒uniaxial compressive strength; *E*_*0*_‒initial elastic modulus.

**Figure 2:**
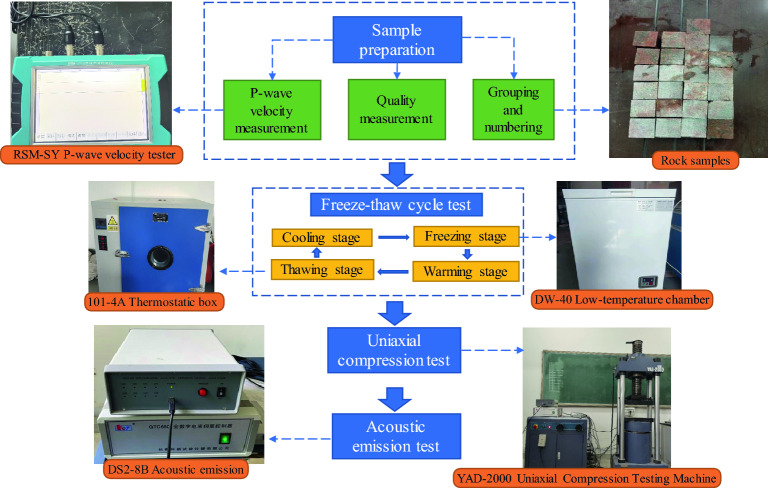
Schematic of the monitoring system and testing process.

The experimental instruments are mainly divided into three parts, the freeze‒thaw cycle system, loading equipment and AE monitoring system, as shown in Fig. 2. A 101-4A high-temperature drying oven and a DW-40 low-temperature oven were used for the freeze‒thaw testing, with a controllable temperature range of − 40 ~ 300 °C. A RSM-SY6 acoustic tester equipped with one transmitting channel and two receiving channels, with a bandwidth of 1–500 kHz, was used to measure the wave velocity of the freeze‒thaw rock samples. The uniaxial compression tests were conducted using a YAD-2000 microcomputer-controlled electrohydraulic servo pressure testing machine made in Changchun, China. The maximum test force of the testing machine is 2000 kN, the effective stroke is 200 mm, and the loading rate is 0–55 mm/min. The DS2-8B multichannel AE system was used to capture the microbreakage signals during the uniaxial compression of mixed granite samples. The main amplifier of the system was incrementally set to 40 dB, the threshold was set to 40 dB, and the sampling rate was 1 MHz. The AE probes used in the testing are model RS-2A, made by Softland Times, with a ceramic contact surface, a frequency range of 50 kHz to 400 kHz, a center frequency of 150 kHz, and an operating temperature of − 20 to 130 °C.

### Experimental procedure

**Figure 3:**
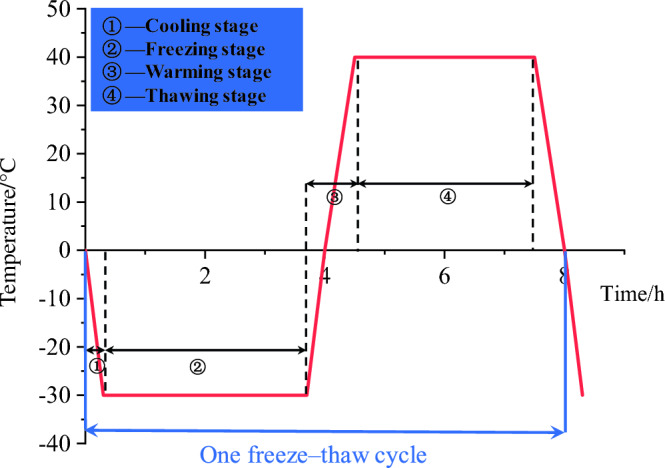
Temperature‒time curve employed in the freeze‒thaw cycle test.

The rock samples were water-saturated before freeze‒thaw cycle tests. Initially, the rock samples were placed in an oven at 105 °C for a duration of 24 h. Subsequently, after 48 h of free water saturation to simulate natural submerged rocks, the rock samples were prepared for the ensuing freeze‒thaw tests. Referring to the temperature conditions of the mining area, the freeze‒thaw cycle temperature was calibrated at − 30 ~ 40 °C. The specific operation is the water-saturated rock samples wrapped in plastic wrap, frozen for 4 h, and then open the plastic wrap to put the rock samples into the thermostat in the sink for melting, melting process lasts 4 h. The freeze‒thaw cycle test process is illustrated in Fig. 3. In this study, five groups of rock samples were subjected to 0, 10, 20, 30, and 40 freeze‒thaw cycles, with each group encompassing 3 valid samples. Notably, for every set of 5 freeze‒thaw cycles, an assessment was conducted to determine the mass and wave velocity of the mixed granite samples before and after the freezing and thawing procedures.

**Figure 4:**
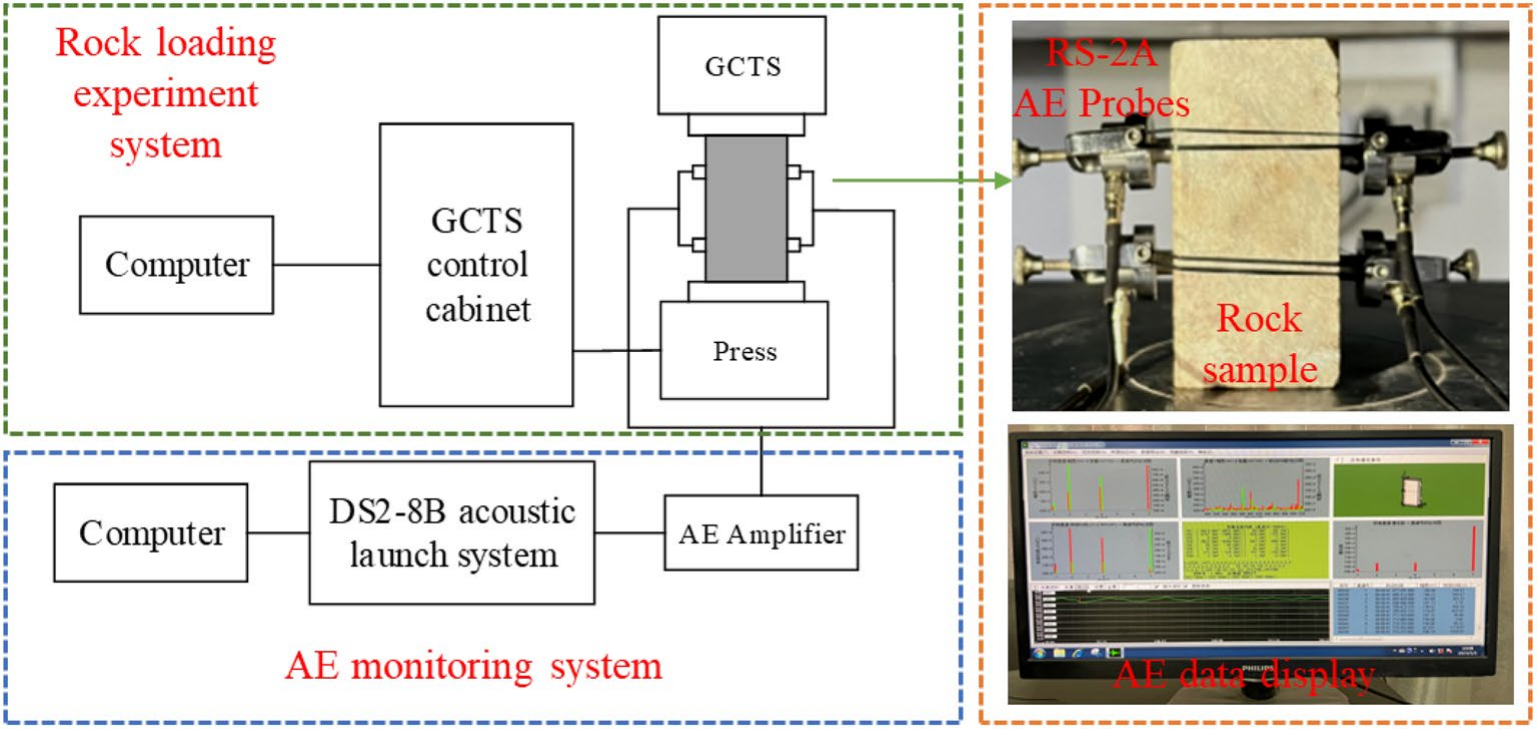
The uniaxial compression test system and AE monitoring system.

Following the freeze‒thaw cycle tests, uniaxial compression tests were carried out on a YAD-2000 testing machine with a loading rate of 0.06 mm/min and a data acquisition frequency of 1 MHz. To obtain the microfracture signals during the compression process, four AE probes were symmetrically arranged from top to bottom on both sides of the rock sample surface. Then, the AE probes were connected to the data acquisition instrument. The uniaxial compression test system and AE monitoring system are shown in Fig. 4.

## Results and discussion

### Variations in physical properties of mixed granite with different numbers of freeze‒thaw cycles

**Figure 5:**
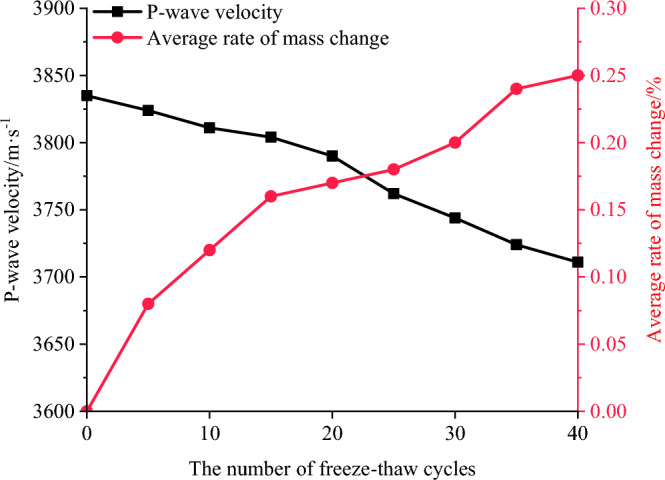
The evolution of the mass change rate and wave velocity of mixed granite with different freeze‒thaw cycles.

As shown in Fig. 5, the mass and wave velocity of water-saturated mixed granite specimens, after varying numbers of freeze–thaw cycles, were experimentally determined. The average mass change rate was calculated using the following Eqs. (1) and (2). *K*_m_ denotes the change in the average mass of the rock sample. The average rate of mass change is calculated by the following equation and can be used as an indication of damage to rock samples under the action of freeze–thaw cycles.

1 $$\overline{{m_{n} }} = {{\left( {m_{1} + m_{2} + m_{3} } \right)} \mathord{\left/ {\vphantom {{\left( {m_{1} + m_{2} + m_{3} } \right)} 3}} \right. \kern-0pt} 3}$$

2 $$K_{m} = \frac{{\overline{m}_{n} - \overline{m}_{0} }}{{\overline{m}_{0} }}$$

where $\overline{m}_{n}$ and $\overline{m}_{0}$ represent the average mass after n freeze–thaw cycles and the initial average mass. $m_{1}$, $m_{2}$, $m_{3}$ is the mass of the rock sample at each set of freeze–thaw times. $K_{m}$ is the average rate of mass change.

During the freeze‒thaw cycles, the water–ice phase transformation in the rock fractures generates frost heave force, which leads to the initiation and expansion of cracks^33^. Concurrently, the proportion of pore water gradually escalates under the influence of external water sources. Consequently, the mass of the rock samples exhibits fluctuations in correspondence with the number of freeze‒thaw cycles, demonstrating an overall upward trajectory. At 20 freeze‒thawing cycles the mixed granite has highly developed internal fissures, a small amount of residue is shed from the surface, and the quality of the rock samples decreases by a small amount, but the mass average rate of change is unchanged due to the recharge of water.

As the number of freeze‒thaw cycles increases, a discernible decreasing trend of longitudinal wave velocity emerges. Notably, this decline becomes more pronounced after the 25th cycle, with a reduction of 1.3%. Subsequently, after 40 cycles, the wave velocity experiences a more substantial reduction of 2.6%, causing the longitudinal wave velocity to shift from 3835 to 3711 m/s. The mass change rate curve shows an S-shaped trend, with a significant increase in mass before 15 freeze‒thaw cycles, a slow increase between 15 and 30 cycles, and a sharp increase after 30 cycles. The results indicate that the deterioration phenomenon inside the rock samples gradually intensifies. Furthermore, this phenomenon alludes to an elevated occurrence of defects, and thus an increased porosity, within the mixed granite specimens.

### Evolution characterization of mechanical properties of mixed granite with different numbers of freeze‒thaw cycles

#### Comparison of stress‒strain curves

**Figure 6:**
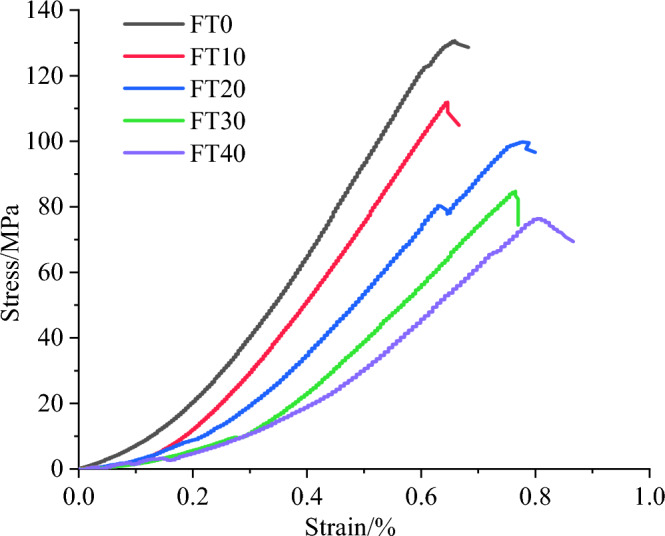
The uniaxial strain‒stress curves of mixed granite with different numbers of freeze‒thaw cycles.

As shown in Fig. 6, the uniaxial compressive stress‒strain curves of mixed granite after different numbers of freeze‒thaw cycles can be divided into four stages: compaction stage, linear elastic stage, nonlinear deformation stage, and postpeak failure stage. As the number of freeze‒thaw cycles increases, the compaction stage is significantly prolonged. Additionally, the stress‒strain curve assumes a flatter trajectory, and a considerable reduction in uniaxial compressive strength is observed. This indicates that the internal joints and fractures of the rock samples further expand during the freeze‒thaw cycles. Simultaneously, the water softening effect also weakens the rock resistance to deformation. The peak strength of the freeze‒thawed rock samples continuously decreases, and the axial strain increases.

#### Mechanical parameters

**Figure 7:**
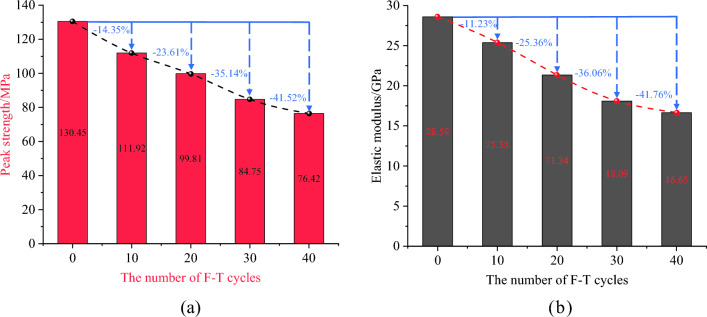
The evolution characteristics of uniaxial compressive strength and elastic modulus with different numbers of freeze‒thaw cycles: (**a**) uniaxial compressive strength and (**b**) elastic modulus.

As shown in Fig. 7, the uniaxial compressive strength and elastic modulus law curves of mixed granite with different numbers of freeze–thaw cycles were obtained. As the number of freeze‒thaw cycles increases, there is a gradual decline in both the compressive strength and elastic modulus. The peak strength of the rock sample after 0 freeze‒thaw treatments (no treatment) is 130.67 MPa. The rates of compressive strength change after 10, 20, 30, and 40 cycles are recorded as 14.35%, 23.61%, 35.14%, and 41.52%, respectively, and ultimately reach a diminished value of 76.42 MPa (Fig. 7a). The results indicate that the compressive strength of mixed granite is greatly affected by freeze‒thaw cycling. Additionally, as shown in Fig. 7, the defects within the rock samples manifest rapid development before 20 freeze‒thaw cycles, corresponding to a rapid decrease in peak strength. Subsequently, the cumulative damage rate of the rock tends to stabilize, and the rate of reduction in the peak strength decreases.

As shown in Fig. 7b, similar to the trend of uniaxial compressive strength, the elastic modulus decreases nonlinearly with the increase in the number of freeze‒thaw cycles. After 40 freeze‒thaw cycles, the elastic modulus decreases from 28.59 to 16.65 GPa, with a decay rate of 41.76%. Notably, the loss rate of the elastic modulus is relatively high before 30 freeze‒thaw cycles, resulting in a decrease of 36.73%. The decrease in the elastic modulus reflects the weakened deformation resistance of the mixed granite. As the number of freeze‒thaw cycles increases, the development of cracks gradually increases, and the deterioration of rock mechanical properties gradually increases. Specific data can be found in Table 2.

**Table 2:** Results of mechanical tests on different samples.

F-T cycles	Specimen name	Maximum peak stress/MPa	Mean value/MPa	Standard deviation	Elastic modulus/GPa	Mean value/GPa	Standard deviation
0	FT-0-1	124.87	130.45	2.82	26.06	28.59	2.57
	FT-0-2	131.12			27.59		
	FT-0-3	135.35			32.12		
10	FT-10-1	103.39	111.92	4.81	20.73	25.38	3.22
	FT-10-2	111.91			25.78		
	FT-10-3	113.88			28.63		
20	FT-20-1	97.59	99.81	1.70	18.29	21.34	1.92
	FT-20-2	100.13			22.56		
	FT-20-3	101.71			22.14		
30	FT-30-1	81.59	84.75	3.85	19.19	18.09	2.07
	FT-30-2	82.50			20.64		
	FT-30-3	90.16			24.14		
40	FT-40-1	74.79	76.42	1.82	16.18	16.68	0.57
	FT-40-2	75.52			16.57		
	FT-40-3	78.96			17.30		

### AE count of mixed granite after different numbers of freeze‒thaw cycles

**Figure 8:**
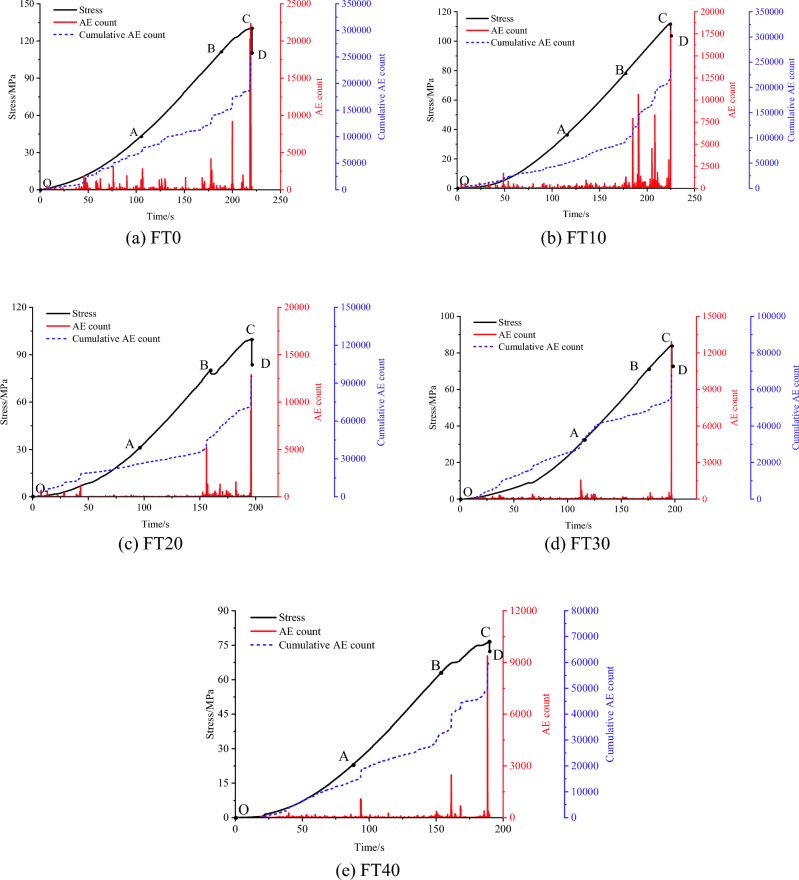
The characteristics of AE counts of granite with the increase in the number of freeze‒thaw cycles.

AE count refers to the tally of ringing pulse oscillations that surpass the established threshold signal within a given timeframe, which to some extent reflects the amplitude of the AE signal. It can reveal the damage characteristics of rock samples caused by the initiation and propagation of microscopic defects. The evolution characteristics of AE counts throughout the loading process of mixed granite samples with different numbers of freeze‒thaw cycles are shown in Fig. 8.

As shown in Fig. 8, the stress‒strain curves of mixed granite after different numbers of freeze‒thaw cycles can be divided into four stages: compression stage (OA), elastic deformation stage (AB), yield stage (BC), and postpeak stage (CD). Moreover, the characteristics of the AE signals during the loading process of the rock sample are basically consistent with those of the stress‒strain curve.

During the compaction stage (OA), both the AE counts and AE cumulative counts increase gradually. This phenomenon stems from the structural modifications within the rock while under loading, generating low-frequency signals that correspond to a limited number of AE events. More AE signals appear in the compaction stage of granite mixed with 30 freezing and thawing cycles, corresponding to a sudden increase in the cumulative ringing counts, which may be attributed to the uneven expansion of local defects in the rock samples under the action of freezing and thawing and the expansion and interaction of local cracks under the action of axial loading, resulting in the enhancement of short-term acoustic signals.

In the elastic stage (AB) and elastic–plastic deformation stage (BC) of the freeze–thaw mixed granite, the number of AE events and the AE cumulative count increased significantly, of which the AE cumulative count increased in a segmental manner. The AE signals are abrupt (at point C), which corresponds to a steep increase in the AE counts at the point of stress drop. When the development of internal defects in the rock is enhanced, the local cracks expand and interpenetrate under loading, and the degree of rock deterioration increases.

Prior to reaching the peak stress, the rock undergoes the gradual formation of a fracture network. Local fractures intersect and interpenetrate, leading to the frequent appearance of microfracture characteristics and a corresponding significant enhancement in AE signals. Additionally, there is a noticeable period with a lack of AE signals before damage occurs in the mixed granite after undergoing freeze‒thaw cycling. During this period, there is no significant AE activity, but the elastic strain energy continues to accumulate.

During the destructive stage (CD), the previously accumulated elastic strain energy is promptly released. This results in an abrupt jump in the deformation of the rock sample, a rapid decline in stress levels, and the formation of macroscopic cracks. Consequently, the mixed granite specimen moves toward complete destruction. Notably, the AE cumulative count continues to surge.

Furthermore, notable discrepancies arise in the evolutionary traits of both the AE count and AE cumulative count within mixed granites subjected to varying numbers of freeze‒thaw cycles. The overall AE cumulative count exhibits a decrease as the number of freeze‒thaw cycles increases. This phenomenon could be attributed to the expansion of microfissures and the inception of new fissures due to freeze‒thaw cycles. This process, in turn, leads to localized sample deterioration and diminishing cohesion. Consequently, energy release takes place earlier during the uniaxial compression process. Since AE monitoring commences at the onset of uniaxial compression, it does not encompass the freeze‒thaw cycle stage. Additionally, the AE activities within the mixed granite samples without freeze‒thaw treatment manifest as frequent with a wide distribution time range. This pattern suggests nonuniformly distributed defects within the mixed granite, implying a progressive degradation of the rock samples. AE signals cluster notably after the 10th and 20th freeze‒thaw cycles, accompanied by a reduction in AE cumulative count. Nonetheless, a sudden increase in the localized AE count occurs immediately prior to the stress peaks. This effect narrows the accumulation range, aligning closely with the peak strain point. In the context of the 30th and 40th freeze‒thaw cycles, AE signals become increasingly frequent and relatively dispersed. The prevalence of microrupture signals and low-level fluctuations in AE count characterize this stage. Notably, during the prepeak elastic‒plastic deformation phase, the AE cumulative count exhibits a sharp rise. This phenomenon suggests a transformation in the internal structure of the samples, signifying a shift from brittle to ductile damage progression. Ultimately, the samples undergo complete deterioration.

### AE amplitude and frequency characteristics of mixed granite after different numbers of freeze‒thaw cycles

The amplitude and peak frequency, as the main parameters of AE characteristics, can reflect the fracture information of mixed granite. The main AE frequency distribution of mixed granite ranges from 0 to 120 kHz. According to its distribution characteristics, the main frequency is divided into three bands, 0–40 kHz, 40–80 kHz, and 80–120 kHz, which are defined as low frequency (LF), intermediate frequency (IF), and high frequency (HF). Correspondingly, the amplitude variation range is also divided into two groups, 60–75 dB and 75–90 dB, defined as low amplitude (LA) and high amplitude (HA). Among them, low-amplitude signals are in the low-, middle- and high-frequency bands, while high-amplitude signals are only in the low-frequency band. Therefore, AE frequency signals can be categorized into high-frequency-low-amplitude (HF-LA), intermediate-frequency-low-amplitude (IF-LA), low-frequency-low-amplitude (LF-LA), and low-frequency-high-amplitude (LF-HA) signals. Santis and Tomor^34^ also pointed out that high-amplitude signals are usually concentrated in the low-frequency region, which indicates the release of high energy.

#### Main frequency variation characteristics of AE after 40 freeze‒thaw cycles

**Figure 9:**
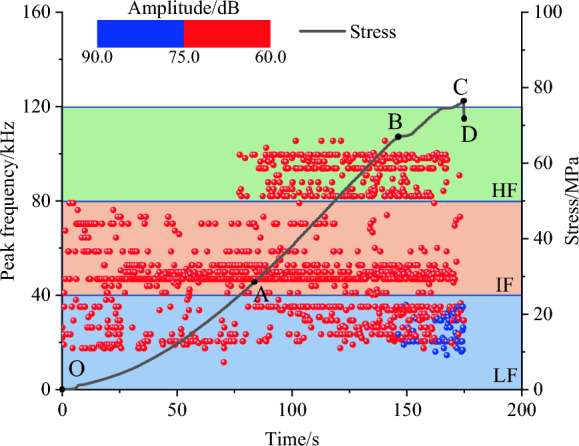
Amplitude-frequency characteristics of AE in FT-40 granite.

The amplitude–frequency characteristics during the fracture process of mixed granite subjected to 40 freeze‒thaw cycles are shown in Fig. 9. Through an examination of the primary frequency of rock AE, Hu^35^ and Wang ^31^ demonstrated that the HF-LA, IF-LA and HF-LA signals correspond to the AE signals of small-scale cracks. The LF-HA signals correspond to the AE signals of medium- and large-scale fissures.

As shown in Fig. 9, taking the case of mixed granite after 40 freeze‒thaw cycles as an example, the amplitude and frequency characteristics of AE are analyzed. (1) In the OA stage, the AE signals are mainly distributed in the intermediate-frequency region, centered between 46.9 and 52.7 kHz, with sporadic frequencies in the low-frequency region. The reason for this phenomenon is that the primary pores and microfractures close in the early stage of loading; the small-scale microfractures predominantly correspond to IF-LA signals. (2) In the AB stage, three frequency bands of AE signals appeared simultaneously: LF-LA, IF-LA and HF-LA. In contrast to the two intermediate-frequency bands of the OA stage, a supplementary frequency band materializes in the high-frequency domain, centered at approximately 93.6 kHz. Concurrently, sporadic AE signals manifest within the low-frequency domain. The coexistence of these three signals denotes a multifaceted internal fracture pattern within the mixed granite during this stage; however, small-scale cracks remain dominant. (3) In the BC stage, four types of AE signals occur simultaneously: LF-LA, IF-LA, HF-LA and LF-HA signals. Significantly, the LF-HA signals appeared for the first time.

The overall AE signals intensify, while the low-frequency signals remain scattered. These observations suggest the continued dominance of small-scale crack expansion within the mixed granite during this stage. However, there is also small-scale crack propagation and coalescence, forming large-scale cracks. (4) In the CD stage, the AE signals decrease after the peak strength. The LF-HA signals predominate, while the LF-LA, IF-LA and HF-LA signals decrease. This means that large-scale cracks (macroscopic fracture surfaces) in rock samples are formed in this stage.

#### Variation characteristics of the AE main amplitude-frequency relationship of mixed granite after different numbers of freeze‒thaw cycles

**Figure 10:**
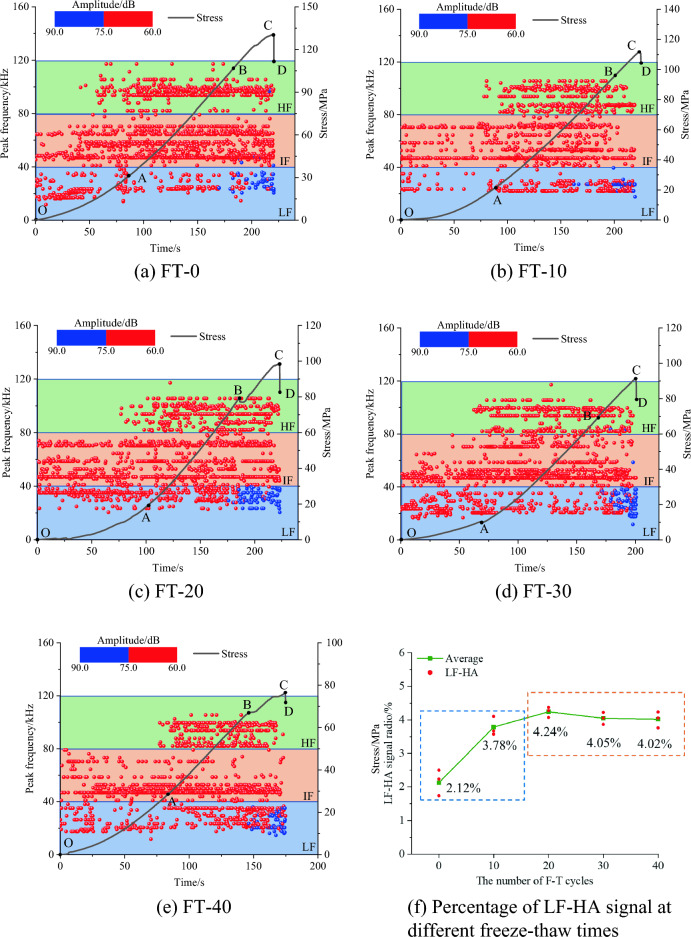
Variation characteristics of the AE main frequency-amplitude of relationship after different numbers of freeze‒thaw cycles.

As shown in Fig. 10, the main frequency amplitude of the AE from mixed granite samples subjected to varying numbers of freeze–thaw cycles differs significantly from those not subjected to such cycles. Specifically, after 10 freeze–thaw cycles (Fig. 10b), the AE signals become more dispersed compared to the unfrozen mixed granite. In the OA region, signals scatter, the number of AE events decreases, and during the AB period, three distinct signals emerge, with a reduction in the number of signals per unit of time. This indicates that after undergoing freeze–thaw cycles, initial development of internal fractures occurs within the mixed granite. These fractures are relatively simple under low stress conditions but become increasingly complex as stress levels rise. The overall number of AE events decreases, signifying a weakening in the characteristics of small-scale micro-fractures. During the CD stage, signals predominantly exhibit HF-LA and LF-LA characteristics, accompanied by sporadic MF-LA signals, indicating increased rock damage. Mixed granite samples enduring 20 freeze–thaw cycles exhibit a significantly higher number of AE events compared to those subjected to 10 cycles, suggesting that the freeze–thaw cycles exacerbate sample damage, leading to rapid deterioration. Low amplitude signals were mainly observed between 30 and 50 kHz. LF-HA signals increased and peaked after 20 freeze–thaw cycles, compared to those after 10 freeze–thaw cycles.

The distribution of the main frequency amplitude of AE signals similar when subjected to 30 and 40 freeze–thaw cycles, with the signals concentrated within the 40–60 kHz range. Furthermore, the proportion of LF-HA signals undergoes a slight decrease compared to previous observations. This indicates that the cyclic freeze–thaw process promotes overall degradation of the rock samples, leading to a reduction in large-scale damage resulting from uneven development of defects. Damage predominantly progresses through the expansion and coalescence of microcracks, signifying stable damage within the rock samples.

The ratio of LF-HA signals (as shown in Fig. 10f) can be utilized to assess the presence of large-scale fissures within the samples. With an increasing number of freeze–thaw cycles, the LF-HA AE signals from the rock samples initially increase and then stabilize. The highest number of large-scale cracks is produced after 20 freeze–thaw cycles, after which the overall degradation of the rock samples leads to a reduction in large-scale damage caused by the uneven development of defects.

To offer deeper insight into the microscopic damage mechanisms of rocks subjected to varying numbers of freeze‒thaw cycles, Table 3 presents the ratios of high- and low-frequency signals. Notably, an observed increase in low-frequency signals coincides with a decrease in the proportion of high-frequency signals. According to the published literature^36^, due to the natural fractures in the mixed granite rock sample, the intermediate frequency signal characterizing microcrack friction has existed since the AB stage. Additionally, the friction between mineral particles or intercrystalline ruptures gives rise to low-frequency signals. Remarkably, as the number of freeze‒thaw cycles increases, a reduction in the occurrence of ruptures within porous microcracks is observed.

**Table 3:** Proportion of the low- and high-frequency AE signals during rock deformation.

F-T cycle	Ratio of low frequency (%)	Ratio of high frequency (%)
0	14.46	26.66
10	18.58	25.21
20	25.14	23.27
30	25.01	21.26
40	28.61	19.87

## Failure modes based on RA-AF

**Figure 11:**
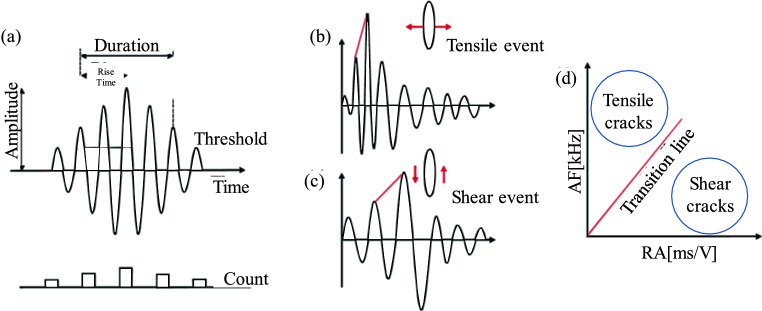
Principle of crack classification: (**a**) calculation parameter, (**b**), (**c**) typical tensile and shear event waveforms, respectively, and (**d**) classification model (modified from Du et al.^41^).

Rock failure is mostly shear failure, tensile failure, or a combination of tensile and shear failure. Numerous studies have demonstrated that the AE parameters RA and AF serve to differentiate between tensile and shear cracks within rocks^37–39^. A depiction of the crack distribution principle is presented in Fig. 11. RA and AF can be defined as follows:

3 $$RA = \frac{Rise\,time}{{Amplitude}}$$

4 $$AF = \frac{AE\,count}{{Duration\,time}}$$

where *RA* is the ratio of rise time to amplitude, ms/V; *AF* is AE frequency and can be derived by dividing the AE counts by the duration, kHz.

**Figure 12:**
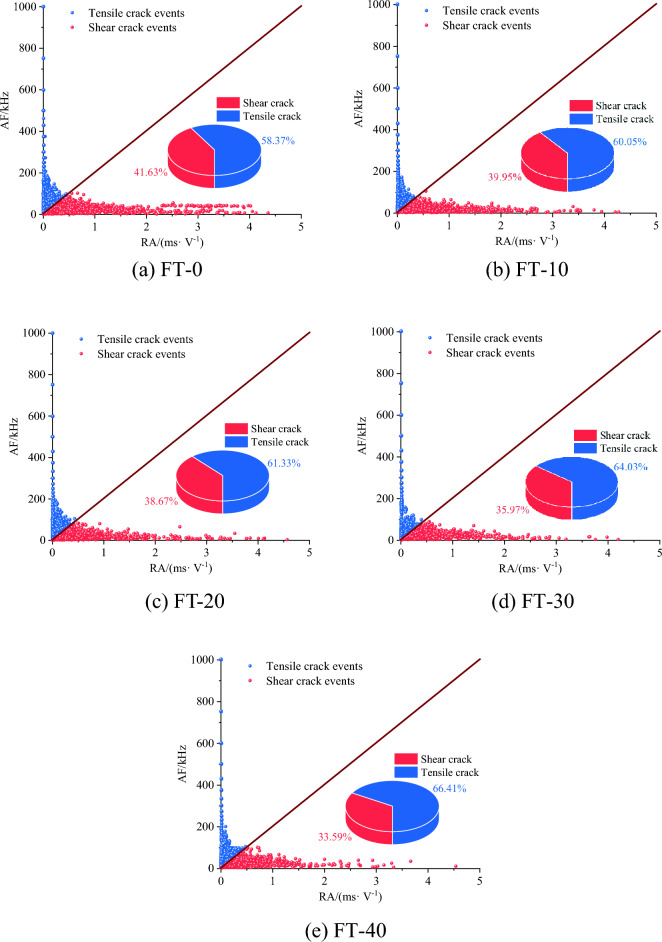
Types and distribution of microcracks in granite under uniaxial compression with different numbers of freeze‒thaw cycles.

According to Eqs. (3) and (4), the RA and AF values of the mixed granite samples after different numbers of freeze‒thaw cycles were calculated, and the distribution diagram of RA-AF was plotted, as shown in Fig. 12. To discern between shear and tensile cracks, a pivotal slope parameter *k* was employed, adopting an RA-AF ratio of 1:200^38,40^. Notably, the AF values are distributed within the range of 0–1000 kHz, while the RA values are distributed within the span of 0–5 ms/V.

According to the results shown in Fig. 12, as the number of freeze‒thaw cycles increases, the internal microcracks of the mixed granite show a decrease in shear cracks and an increase in tensile cracks. This observation aligns with the findings of Du^36^, suggesting that freeze‒thaw-induced rock fractures are chiefly characterized by thoroughgoing fractures and intergranular fractures, among which tensile cracks dominate. Notably, the propagation strength of these cracks remains moderately consistent throughout the loading process. However, with the increase in the number of freeze‒thaw cycles, the crack propagation strength significantly increases.

Among them, the internal tensile microcracks of the mixed granite without freeze‒thaw treatment are dominated by tensile microcracks, and the formation of obvious tensile weak surfaces through multiple cracks causes the rock samples to undergo multicrack tensile damage; the proportion of shear cracks in the mixed granite in the 10th freeze‒thaw cycle decreases, the tensile cracks increase, and the RA-AF point is close to the origin of the coordinates. This result indicates that after 10 freeze‒thaw cycles, the mixed granite is still dominated by tensile cracks, the number of microfractures is increased by the formation of composite cracks, the final damage mode is dominated by tensile cracks, and the amount of mixed damage is greater. After 20 and 30 freeze‒thaw cycles, the internal mixed granite is dominated by tensile and composite fissures, which corresponds to macroscopic tensile-dominated and localized shear damage, and the percentage of shear cracks decreases further. After 40 freeze‒thaw cycles, the mixed granite is dominated by tensile cracks and composite fissures. In this granite, large cracks generally correspond to tensile damage, and small cracks mostly correspond to tensile and shear composite cracks.

### Macroscopic damage patterns of mixed granite

**Figure 13:**
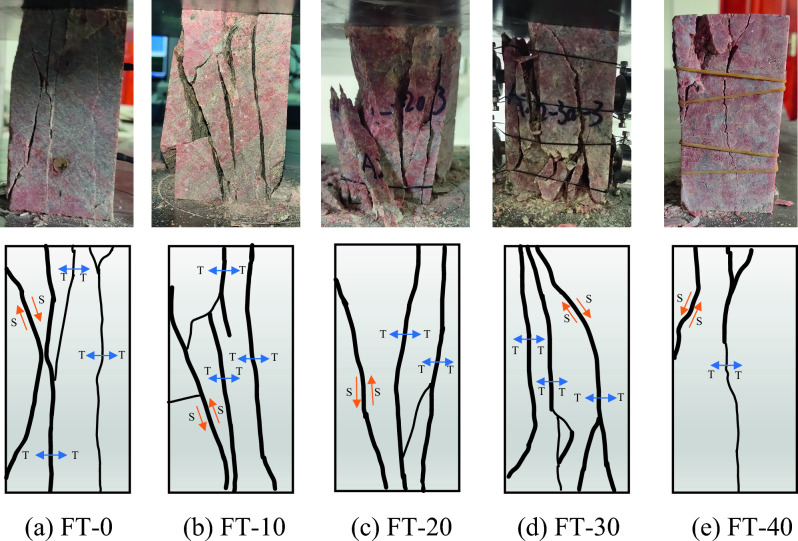
Failure mode and fracture distribution for different numbers of freeze‒thaw cycles.

The observed macroscopic failure pattern serves as a direct reflection of the underlying microscopic processes within the rock. This failure pattern holds considerable significance as a reference for further studying the damage mechanisms of mixed granite samples subjected to freeze‒thaw cycles. The failure pattern and distribution of cracks within the rock samples after different numbers of freeze‒thaw cycles are shown in Fig. 13. In this figure, "T" denotes tensile cracks, while "S" represents shear cracks.

During uniaxial compression tests, it becomes evident that the dominant form of damage within the rock samples is primarily tensile in nature, regardless of the varying number of freezing and thawing cycles. The mixed granite samples exhibit a continuous progression, expansion, and interconnection of tensile cracks. Consequently, the surfaces of the damaged rock exhibit a profusion of tensile cracks, ultimately leading to the generation of numerous fragments.

Under the condition of zero freeze‒thaw cycles, the principal form of damage is characterized by tensile fractures, while shear damage appears to be more sporadic. In contrast, after undergoing 30 cycles of freezing and thawing, the internal damage within the rock samples is notably pronounced. The internal surfaces of damage intersect to form a zone of extensive damage, resulting in a high degree of rock fragmentation.

When a rock specimen undergoes a freeze‒thaw cycle and is subsequently subjected to external loading, the resulting cracks primarily exhibit tensile damage. These cracks typically form along a direction parallel or nearly parallel to the loading direction. The complexity of microfractures that develop within the rock specimen during the freeze‒thaw cycle directly correlates with the complexity of the ultimate damage surface in the rock. As the freeze‒thaw cycle progresses, the formation of each failure surface establishes connections with others, ultimately creating a broader failure zone. It is within this zone that the rock sample eventually fails. In the case of a rock specimen subjected to 20 freeze‒thaw cycles, the inclinations of its natural fractures interact with axial pressure, creating shear cracks. With a further increase in the number of freeze‒thaw cycles, the damage mode gradually shifts toward mixed tensile-shear damage. For rock samples subjected to 40 freeze‒thaw cycles, fewer macroscopic cracks are formed. In this scenario, shear cracks manifest in the upper portion of the cracks, while the middle section displays signs of tensile damage. The extent of damage within the rock samples is relatively modest. This may be due to the fact that the mixed granite samples experienced more freeze–thaw cycles, increased ductility, and decreased peak strength. Consequently, the macroscopic damage transforms from an overall pattern to a localized one.

## Conclusion

This study mainly focuses on the mechanical behavior and failure mechanism of mixed granite on a slope of an open-pit mine in Northeast China after different numbers of freeze‒thaw cycles. Through freeze‒thaw cycle tests and uniaxial compression tests, some key physical and mechanical properties of the rock samples were measured, such as the changes in mass, wave velocity, and strength, the stress‒strain curve, and the AE characteristics. Through a multifaceted approach, the study conducted a detailed analysis of the evolution of freeze–thaw damage to reveal the destabilization and damage patterns of the mixed granite under different freeze–thaw cycles, resulting in the following conclusions:

1. The mass, longitudinal wave velocity, uniaxial compressive strength and elastic modulus of the mixed granite samples decreased with the increase in the number of freeze‒thaw cycles. The wave velocity decreased from 3835 to 3711 m/s; the uniaxial compressive strength decreased from 130.76 to 76.42 MPa; and the elastic modulus decreased from 28.59 to 16.65 GPa.
2. Four stages were labeled to study the AE counts of mixed granite under freeze‒thaw cycles, where the AE signals were silent and then gradually active, reaching a maximum after a brief disappearance prior to damage. In addition, the total cumulative AE counts decrease as the number of freeze‒thaw cycles increases, representing an increase in the ductility of the rock samples, with sufficient internal deterioration and increased damage.
3. During the fracturing process of mixed granite with different numbers of freeze‒thaw cycles, there are four types of AE signals with different frequency and amplitudes recorded: LF-LA, IF-LA, HF-LA and LF-HA. The LF-HA signals corresponding to large-scale macroscopic cracks first increase to 4.24%, and then slightly decrease after 20 freeze–thaw cycles before stabilizing. This phenomenon signifies that after 20 freeze‒thaw cycles, an increase in large-scale fractures transpires, preceding an overall deterioration of the rock samples. Moreover, this decrease in large-scale damage can be attributed to the attenuation of unevenly developed defects. In addition, by analyzing the proportion of high and low frequency signals under different freeze–thaw cycles, it was found that low frequency signals gradually increased and high frequency signals gradually decreased. It means that as the number of freeze–thaw cycles increases, the activity of transgranular or intergranular cracks becomes more intense, while microcrack fracture decreases.
4. The RA-AF values of AE and the fracture mode of rock samples indicate that tensile failure is dominant under different freeze–thaw cycles. As the number of freeze–thaw cycles increases, shear cracks gradually decrease.

## Data Availability

All data generated or analyzed during this study are included in this published article.
